# What Drives Elderly People in China Away from COVID-19 Information?

**DOI:** 10.3390/ijerph19159509

**Published:** 2022-08-02

**Authors:** Xudong Gao, Feng Ding, Ting Ai

**Affiliations:** 1 Department of Nursing, College of Medicine and Health Science, Wuhan Polytechnic University, Wuhan 430023, China; gaoxudonglou@163.com; 2 Library and Information Science, School of Information Management, Wuhan University, Wuhan 430072, China; 3 Psychological Counseling Center, College of Medicine and Health Science, Wuhan Polytechnic University, Wuhan 430023, China; aiting2050@163.com

**Keywords:** COVID-19, elderly people, information avoidance, information overload, health information

## Abstract

*Background*: A worrying phenomenon has emerged in recent years: a growing number of people have stopped seeking coronavirus disease 2019 (COVID-19) information and have started deliberately avoiding it. Even though the virulence of COVID-19 has now weakened, the proportion of severe illnesses and deaths in elderly people is still much higher than in other age groups. However, no study has focused on this topic. This is the first study to explore the level of COVID-19 information avoidance among elderly people, and to identify the barriers and potential factors associated therewith. *Methods*: Convenience sampling was used to recruit 907 elderly people in Wuhan, China. Data collection measures included a sociodemographic questionnaire, health information avoidance scale, information overload scale, general self-efficacy scale, and health anxiety inventory. *Results*: A total of 72.3% of elderly participants reported COVID-19 information avoidance. Regarding COVID-19-related information reading habits, 44.5% of the elderly only read the title, 16.0% merely skimmed through the content, and 22.9% skipped all relevant information. The most common reasons for this result were information overload (67.5%), underestimation of the infection risk (58.1%), and uselessness of information (56.4%). The main factors associated with COVID-19 information avoidance were recorded as information overload, age, health anxiety, and children (*p* < 0.05). *Conclusions*: China should strengthen its health communication regarding COVID-19 in accordance with the characteristics of elderly people, adopt more attractive publicity methods on traditional media, improve censorship about health information, and pay more attention to the childless elderly and the elderly aged 80 and above.

## 1. Introduction

The exchange and use of health information is an important component of health services and is one of the six cornerstones of countries’ health systems proposed by the World Health Organization (WHO) [1,2,3]. Health information contributes to individuals’ understanding of medical policies, expands their knowledge of health, and improves their health behaviors [4,5,6,7]. The coronavirus disease 2019 (COVID-19) was first reported in Wuhan, the largest city in central China, in December 2019 [8]. Initially, due it being highly contagious, its dangerous complications, and the dearth of effective treatments, the public was often eager to obtain as much information about COVID-19 as possible [9,10]. That information helped many people keep abreast with the latest progress of the pandemic, strengthened self-protection awareness, and reduced the risk of infection [11,12]. Over the past two years, people around the world were flooded by a great amount of COVID-19-related information, resulting in some opting not to receive information about COVID-19 anymore [13]. In other words, a worrying phenomenon has emerged: a growing number of people are stopped seeking COVID-19 information and are now deliberately avoiding it [14].

Health information avoidance refers to individuals consciously eschewing available health information or procrastinating in obtaining it [15]. Health information avoidance generally exhibits the following characteristics: First, the health information in question can be readily obtained; people are not precluded from obtaining it due to restrictive conditions. Second, choosing to avoid relevant health information is a deliberate behavior [16,17]. The behavior of health information avoiding is sometimes aimed at a wide range of health information, and other times at a specific disease [18,19]. Health information avoidance might temporarily relieve negative emotions such as anxiety, fear, and sadness, but it could lead to hazardous results in the long run because it deprives people of the opportunity to learn about concomitant risks and to take precautionary measures [16,18,20]. It has been proved that people with higher COVID-19 information avoidance tendencies were less likely to perform protective actions, such as being vaccinated, maintaining safe distances, and wearing masks [21]. Since the first quarter of 2022, more and more countries have canceled anti-pandemic measures. As a result, the public potentially further underestimate the pandemic, and the phenomenon of COVID-19 information avoidance might escalate across the globe. In addition, COVID-19 vaccine hesitancy among elderly people might also affect their COVID-19 information avoidance. Since the COVID-19 vaccine is one of the fastest vaccines ever developed, people in many countries have expressed their skepticism about its efficacy, safety, and side effects [22,23,24]. The abundance of negative information about COVID-19 vaccines might also exacerbate COVID-19 information avoidance.

The elderly usually have weaker immune systems and suffer more from basic diseases compared with younger people. It should not be overlooked that even though the virulence of COVID-19 has now weakened, the proportion of severe illnesses and deaths in elderly people is still much higher than in other age groups [25]. For example, in the first quarter of 2022, about 5100 people in Hong Kong died of COVID-19, 95% of whom were at least 60 years old [25]. In addition, the physical and psychological health of elderly people are relatively fragile, and they are more accustomed to avoiding health information [26]. COVID-19 information avoidance among the elderly should therefore be taken seriously. However, previous studies on COVID-19 information avoidance has mainly focused on populations at large, college students, and consumers [14,27,28,29,30,31]. No studies, to the best of our knowledge, have been undertaken on COVID-19 information avoidance among elderly people. Thus, our study aimed to fill this gap. It is hoped that our research will provide an opportunity to encourage more people to pay attention to COVID-19 information avoidance.

## 2. Methods

### 2.1. Study Design and Participants

COVID-19 information avoidance involves disciplines such as preventative medicine, information management, and psychology. Accordingly, we formed a multidisciplinary team to explore the level of COVID-19 information avoidance among elderly people in China and identified the barriers and potential factors associated with it. We investigated sociodemographic characteristics and the scale of health information avoidance, information overload, general self-efficacy, and health anxiety inventory in the elderly. A cross-sectional survey was launched using paper questionnaires. Regarding data collection availability and quality, elderly people from 14 communities in Wuhan were selected by convenience sampling to participate. A social worker was chosen in each community as our primary point of contact. Questionnaires were distributed to elderly participants by social workers. The social workers provided informed consent forms to the participants prior to the interview. Each participant was fully informed of the purpose and significance of the research and were assured that their participation was both voluntary and anonymous.

Inclusion criteria were participants who, (1) were at least 60 years old, (2) were cognizant and able to read and write, (3) had lived in Wuhan for more than one year, and (4) had volunteered to take part in this study. Exclusion criteria were participants who, (1) were patients who suffered from mental illnesses or cognitive impairments, (2) were unable to obtain any information about COVID-19, and (3) were infected with COVID-19. This study was approved by the Institutional Review Board of Wuhan Polytechnic University (BME-2022-1-03). A total of 1003 elderly people completed the questionnaire during March 2022. Ninety-six questionnaires lacking indispensable information were excluded, and 907 questionnaires were finally analyzed.

### 2.2. Survey Questionnaire

#### 2.2.1. Sociodemographic Characteristics

Sociodemographic characteristics included gender (male, female), age group (60–69 years, 70–79 years, more than 79 years), education level (primary school, middle school, university), employment status (employed, retired), marital status (married, divorced or widowed, single), have children or not (have children, have no children), place of residence (own home, nursing home, other locations), religion (nonreligious, religious), history of COVID-19 vaccination (unvaccinated, vaccinated), and monthly income (less than USD 620, USD 620–USD 1240, more than USD 1240). Monthly income in this survey was recorded in Chinese currency (less than 4000 RMB, 4000–8000 RMB, more than 8000 RMB) and converted to U.S. dollars for reporting purposes.

#### 2.2.2. Health Information Avoidance Scale (HIAS)

The HIAS was developed by Shuai in 2020 [32]. HIAS included three parts: negative emotions (4 items), cognitive conflict (3 items), and behavioral changes (3 items). All items were answered on a 5-point Likert scale ranging from 1 (totally disagree) to 5 (totally agree). The Cronbach’s α coefficient of the HIAS was 0.951. The validity index was 0.738, and the reliability index was 0.871. The scores on the 10 items were aggregated to obtain the total score; higher total scores indicated higher levels of avoidance of COVID-19 information. A HIAS score of 25 or higher indicated COVID-19 information avoidance. Furthermore, the reading habits of elderly people regarding COVID-19 information were also investigated.

#### 2.2.3. COVID-19 Information Overload Scale (CIOS)

The CIOS was developed by Yang in 2021 to assess the extent to which COVID-19 information is more than an individual can accept and process [33]. Participants were asked to answer seven questions to evaluate their COVID-19 information overload on a 5-point Likert scale (0 = none at all, 1 = almost none, 2 = sometimes, 3 = often, 4 = always). Cronbach’s α coefficient of the CIOS was 0.863. The validity index was 0.724, and the reliability index was 0.815. The scores on the seven items were added together to obtain the total score; higher total scores revealed more severe levels of COVID-19 information overload.

#### 2.2.4. General Self-Efficacy Scale (GSES)

The GSES was developed by Scheler in 1982 and translated into a Chinese version by Wang in 2001 [34,35]. Participants were asked to answer 10 questions to evaluate their self-efficacy on a 4-point Likert scale ranging from 1 (totally disagree) to 4 (totally agree). The Cronbach’s α coefficient of the CIOS was 0.870. The validity index was 0.820, and the reliability index was 0.847. The higher total scores revealed better self-efficacy.

#### 2.2.5. Chinese Version of the Health Anxiety Inventory (CHAI)

The Health Anxiety Inventory (HAI) was developed by Salkovskis in 2002, and the Chinese version, the CHAI, was developed by Zhou in 2017 [36,37]. CHAI was used to assess people’s concerns and anxiety about their health. The CHAI comprises two parts: health anxiety (14 items), and risk factors (4 items). All items were answered on a 4-point Likert scale ranging from 0 (totally disagree) to 3 (totally agree). The Cronbach’s α coefficient of the CHAI was 0.864. The validity index was 0.788, and the reliability index was 0.790. The scores on the 18 items were added up to obtain the total score. The higher the total scores, the higher the anxiety level about health.

### 2.3. Statistical Methods

The IBM SPSS Statistics 27.0 (IBM, Chicago, IL, USA) was applied for statistical analysis. The result of the Kolmogorov–Smirnov showed that HIAS was reflected a normal distribution. Multicollinearity was measured by the variance inflation factor (VIF). After testing, values of VIF were all lower than 10, so there was no multicollinearity. Pearson correlation analysis was, respectively, used to examine the correlations between HIAS, as well as COVID-19 information overload, general self-efficacy, and health anxiety. The comparison of different sociodemographic characteristics of the elderly’s COVID-19 information avoidance was analyzed by univariate analysis. Multiple-factor analysis of HIAS was analyzed using multiple regression analysis. For all tests, values of *p* < 0.05 were considered statistically significant.

## 3. Results

### 3.1. Sociodemographic Characteristics of the Participants

A total of 907 qualified questionnaires were obtained. In total, 53.1% of the participants were female, 46.0% were 60–69 years old, *47.2*% were educated in primary school, 84.0% were retired, 68.8% were married, 79.8% had children, 78.6% were nonreligious, and 72.1% were vaccinated. A total of 49.0% of the participants’ monthly income was less than USD 620. In addition, 77.8% of the participants lived in their own homes.

### 3.2. Avoidance of COVID-19 Information among Elderly People

**Figure 1:**
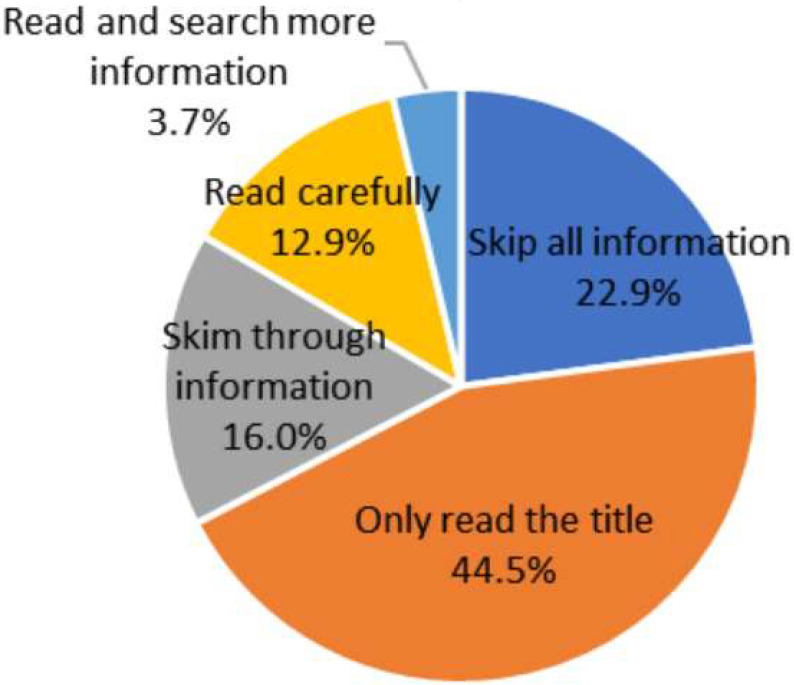
Reading habit of COVID-19 information among elderly people in Wuhan, China (*n* = 907).

The results of COVID-19 information avoidance among elderly people are listed in Table 1. The average HIAS score was 30.64 ± 7.70. The maximum score of HIAS was 42, and the minimum was 13. Furthermore, 72.3% of elderly people scored at least 25 points on the HIAS. The reading habits of COVID-19 information are shown in Figure 1; 44.5% of the elderly only read the title, 22.9% skipped all relevant information, and 16.0% merely skimmed through the content. As shown in Table 2, the reasons for avoiding COVID-19 information were information overload (67.5%), underestimating the infection risk (58.1%), and uselessness of information (56.4%)

### 3.3. COVID-19 Information Overload among Elderly People

The average COVID-19 information overload score among elderly people was 19.12 ± 5.10, with an average accuracy rate of 68.3%. The maximum score of COVID-19 information overload was 27, and the minimum was 11. The COVID-19 information overload score was positively correlated with the HIAS score (*p* < 0.01).

### 3.4. Self-Efficacy of the Elderly

The average self-efficacy score among the elderly was 26.06 ± 5.53, with an average accuracy rate of 65.1%. The maximum score for self-efficacy was 35, and the minimum was 12. No significant correlation was found between the self-efficacy score and HIAS score (*p* > 0.05).

### 3.5. Health Anxiety among the Elderly

The average health anxiety score among the elderly was 30.37 ± 5.79, with an average accuracy rate of 56.2%. The maximum score of COVID-19 information overload was 41, and the minimum was 16. The health anxiety score was positively correlated with the HIAS score (*p* < 0.01).

### 3.6. Comparison of Different Sociodemographic Elderly People’s COVID-19 Information Avoidance

As shown in Table 3, COVID-19 information avoidance among the elderly was significantly associated with their age, employment status, children, and history of COVID-19 vaccination (*p* < 0.05).

### 3.7. Multiple-Factor Analysis of COVID-19 Information Avoidance among Elderly People

As indicated in Table 4, the main factors associated with elderly people’s COVID-19 information avoidance were information overload, age, health anxiety, and children. Specifically, the negative factors included receiving too much COVID-19 information, higher age, anxiety about their health, and childlessness.

## 4. Discussion

Considering the relatively fragile physical and psychological conditions of the elderly, they are still at a high risk in respect to the COVID-19 pandemic [27]. The phenomenon of COVID-19 information avoidance among elderly people can no longer be ignored. This is the first study to explore COVID-19 information avoidance among elderly people, and we submit some targeted recommendations on the subject.

### 4.1. COVID-19 Information Avoidance among Elderly People

Typically, a HIAS score of 25 or higher indicated COVID-19 information avoidance [32]. In this study, a total of 72.3% of the elderly scored at least 25 points on the HIAS, indicating that a large proportion of Chinese elderly people are avoiding information about COVID-19.

We inferred three reasons that might explain this phenomenon. First, it is possible that some elderly people underestimated the risk of COVID-19. By implementing strict border controls, contact tracing, and locking communities, the number of COVID-19 infections in China has been relatively low since May 2020 [38]. Therefore, elderly people in China generally believed that their country was very safe. In addition, the Omicron variant has swept the world since the end of 2021, but the symptoms of infected people were relatively mild and the mortality was close to influenza [39]. For the above reasons, many elderly people thought it was unnecessary to pay attention to relevant information. Second, pandemic prevention and control measures in China might increase avoidance of COVID-19 information. China, a country of 1.4 billion people, has always taken strict pandemic prevention and control measures to ensure that the COVID-19 infection rate remained low and to minimize the death toll [38]. However, China paid a heavy price. Some old people suffered negative consequences, such as food shortages, community lockdown, unemployment, and difficulties in accessing medical services [40]. Some elderly people resisted those inconveniences. It follows that those elderly people might turn their resistance to COVID-19 control measures into resistance to COVID-19 information. Third, news fatigue may also affect older people’s COVID-19 information avoidance. Unlike young people who mainly obtain information through social networks and short videos, elderly people mainly obtain information through traditional media such as television, newspapers and the radio. The news reports of traditional media in China are often serious and homogeneous. COVID-19 first broke out in Wuhan, China, so the local elderly had to watch a large amount of relevant, serious news. It is therefore speculated that COVID-19 news fatigue among the elderly caused them to avoid COVID-19-related information.

### 4.2. Factors Associated with COVID-19 Information Avoidance among Elderly People

This study demonstrated that the main factors associated with elderly people’s COVID-19 information avoidance were information overload, higher age, health anxiety, and children. First, COVID-19 information avoidance among elderly people was closely related to information overload. In general, long-term, massive, and indistinguishable health information would have many effects on recipients, among which information overload is one of the most common results [33]. In our survey, 67.5% of the respondents believed that they received too much COVID-19 information, and 24.6% held the view that it was difficult to distinguish facts from false information. Wang’s study confirmed that health information overload would cause elderly people to employ defensive, psychological mechanisms, and deliberately constrain their access to health information [26]. Therefore, COVID-19 information overload might lead to information avoidance among the elderly. Second, people in the higher age groups were more likely to avoid COVID-19 information. Previous studies found that health information avoidance behavior would increase with age [41]. Older people tend to have lower levels of education, with concomitant lower health awareness, and they are therefore more likely to ignore relevant information. Furthermore, death is regarded as a taboo topic among Chinese, and people tend to avoid talking or thinking about death [8]. Generally, older people tend to be close to death than people in other age groups. Several respondents over the age of 79 claimed that when reading COVID-19 information, they would associate it with death, causing strong death anxiety. Therefore, the death anxiety caused by COVID-19 information might also trigger information avoidance among the elderly. Third, we found that health anxiety of elderly people was closely related to their COVID-19 information avoidance behavior. The elderly tend to be more vulnerable and sensitive to health information [41]. In our survey, 45.4% of the respondents believed that receiving COVID-19 information would cause negative emotions, such as fear, anxiety and sadness. Therefore, the elderly who were particularly concerned about their health, were more likely to avoid COVID-19 information to reduce their negative feelings. Finally, we found that elderly people with children were more receptive to COVID-19 information than those without children. Gong’s study also confirmed that intergenerational support could alleviate elderly people’s sense of powerlessness in the information age and partially reduce elderly people’s health information avoidance behavior [42]. When the Chinese elderly retire, their social circle shrinks and their social activities gradually decrease. The social circle of elderly people in China is basically family-centered, so their children usually become the main COVID-19 information providers. Thus, childless old people may be more likely to avoid COVID-19 information.

### 4.3. Recommendations

Considering these findings, we submit the following suggestions: First, in addition to publicity for the general population, health communication on COVID-19 targeting the specific characteristics of elderly people should be improved. In particular, the childless elderly and the elderly over the age of 80 should be paid more attention to. Second, in view of COVID-19 information fatigue among elderly people, more attractive publicity methods such as humorous talk shows, lively songs, and funny skits can be adopted on traditional media to help people easily absorb relevant knowledge. Third, due to the low educational level and information literacy among the aged, the elderly in China often display a poor ability to distinguish between factual and false health information. Therefore, the government should strengthen censorship about health information to reduce the spread of false or ambiguous COVID-19 information.

### 4.4. Limitations

This study has several limitations. First, due to limited research funds, we only carried out the survey in Wuhan. To solve this difficulty, we plan to cooperate with scholars from other cities to investigate more regions in further research. Nevertheless, Wuhan is the first city in the world to witness the outbreak of COVID-19, and the survey of local citizens was very representative. Second, as a cross-sectional survey, this survey could only evaluate the level of COVID-19 information avoidance at a specified time without follow-up observations of the elderly participants. Third, the HIAS used standardized questions and Likert scores, which facilitated data analyses. However, it also concealed the heterogeneity of COVID-19 information avoidance among elderly people. We therefore intend to carry out in-depth interviews for further exploration in future research. Fourth, methods and scales of measuring COVID-19 information avoidance in studies published on the same topic, but with other populations, are completely different. Therefore, we did not compare our findings with other studies.

## 5. Conclusions

Health information avoidance has often been overlooked in previous research on responses to viral outbreaks. To the best of our knowledge, this study is the first to focus on COVID-19 information avoidance among elderly people in China. A total of 72.3% of elderly participants reported COVID-19 information avoidance. Regarding the reading habits of COVID-19-related information, 44.5% of the elderly only read the title, 16.0% merely skimmed through the content, and 22.9% skipped all relevant information. The most common reasons for this result were information overload (67.5%), underestimating the infection risk (58.1%), and uselessness of information (56.4%). The main factors associated with COVID-19 information avoidance were recorded as information overload, age, health anxiety, and children. Consequently, we emphasize the need to develop measures to counteract COVID-19 information avoidance around the world. The Chinese Government should strengthen its health communication on COVID-19 in accordance with the characteristics of the elderly, adopt more attractive publicity methods on traditional media, improve censorship about health information, and pay more attention to the childless elderly and the elderly aged 80 and above.

## Figures and Tables

**Table 1:** COVID-19 information avoidance among elderly people in Wuhan, China (*n* = 907).

Item	Total Points ($\bar{X}$ ± *S*)	Average Points of Items (Mean ± S)	Score Rates (%)
HIAS	30.64 ± 7.70	3.06 ± 0.77	61.28
Negative emotions	12.28 ± 4.12	3.07 ± 1.03	61.40
Cognitive conflict	8.65 ± 2.26	2.88 ± 0.75	57.67
Behavioral changes	9.72 ± 2.40	3.24 ± 0.80	64.80

Annotation: HIAS = Health Information Avoidance Scale.

**Table 2:** Reasons for COVID-19 information avoidance among elderly people in Wuhan, China (*n* = 907).

Reason	*n*	%
COVID-19 has been well controlled in China and there is no need to pay attention to related information.	527	58.1
COVID-19 information can cause negative emotions, such as fear, anxiety and sadness.	412	45.4
I was overwhelmed with too much COVID-19 information.	612	67.5
Too much false COVID-19 information, which is difficult to distinguish.	223	24.6
Most of the COVID-19 information is useless to me.	512	56.4
I have been vaccinated against COVID-19, so I feel safe.	285	31.4
Other	82	9.0

**Table 3:** Comparison of different sociodemographic elderly people’s COVID-19 information avoidance (*n* = 907).

Factor	*n* (%)	HIAS Total Points ($\bar{X}$ ± S)	*F*	*p*
Gender			3.090	0.079
Male	425 (46.9)	30.16 ± 7.50		
Female	482 (53.1)	31.06 ± 7.85		
Age			55.901	0.000
60–69	417 (46.0)	27.96 ± 8.21		
70–79	331 (36.5)	32.35 ± 6.33		
>79	159 (17.5)	34.14 ± 6.42		
Education level			1.911	0.149
Primary school	428 (47.2)	30.45 ± 7.93		
Middle school	392 (43.2)	31.12 ± 7.13		
University	87 (9.6)	29.46 ± 8.83		
Employment status			7.195	0.007
Employed	145 (16.0)	29.08 ± 7.93		
Retired	762 (84.0)	30.94 ± 7.62		
Marital status			1.886	0.152
Married	624 (68.8)	30.75 ± 8.15		
Divorced or widowed	227 (25.0)	30.83 ± 6.54		
Single	56 (6.2)	28.71 ± 6.52		
Have children or not			39.281	0.000
Have children	724 (79.8)	29.85 ± 7.22		
Have no children	183 (20.2)	33.77 ± 8.68		
Monthly income			1.813	0.164
<USD 620	444 (49.0)	31.04 ± 8.00		
USD 620–USD 1240	308 (34.0)	30.55 ± 6.97		
>USD 1240	155 (17.1)	29.69 ± 8.13		
Place of residence			2.223	0.109
Own home	706 (77.8)	30.47 ± 7.15		
Nursing home	107 (11.8)	30.37 ± 8.68		
Other locations	94 (10.4)	32.22 ± 10.02		
Religion			1.738	0.188
Nonreligious	713 (78.6)	30.47 ± 7.61		
Religious	194 (21.4)	31.29 ± 7.99		
COVID-19 vaccination			4.842	0.028
Unvaccinated	253 (27.9)	31.55 ± 6.26		
Vaccinated	654 (72.1)	30.29 ± 8.16		

Annotation: HIAS = Health Information Avoidance Scale.

**Table 4:** Multiple-factor analysis of COVID-19 information avoidance among elderly people in Wuhan, China (*n* = 907).

Independent Variable	Regression Coefficient	Standardized Regression Coefficient	*t*	*p*
Constant	7.658	-	5.623	<0.001
COVID-19 information overload	0.381	0.253	8.153	<0.001
Age	2.122	0.205	6.957	<0.001
Health anxiety	0.289	0.217	6.989	<0.001
Have children or not	2.733	0.143	4.979	<0.001

Annotation: R^2^ = 0.275, adjusted R^2^ = 0.272, F = 85.534, *p* < 0.05.

## Data Availability

The data that support this study cannot be publicly shared due to ethical or privacy reasons and may be shared upon reasonable request to the corresponding author if appropriate.
